# Surgical Treatment of Congenital Kyphosis in Children: Report of a Rare Complication of Remote Cerebellar Haemorrhage

**DOI:** 10.7759/cureus.56488

**Published:** 2024-03-19

**Authors:** Eleni Pappa, Anastasia Pilichou, Spyridon I Antonopoulos, Spyridon Maris, Konstantinos Neroutsos, Savvas Melissaris, Panagiotis Krallis

**Affiliations:** 1 Orthopaedics, KAT General Hospital, Athens, GRC; 2 Orthopaedics and Trauma, Paediatric Orthopaedics, Aghia Sophia Children's Hospital, Athens, GRC; 3 Orthopaedics & Trauma, Hellenic Red Cross General Hospital, Athens, GRC; 4 Orthopaedics, Aghia Sophia Children's Hospital, Athens, GRC; 5 Neurosurgery, General Hospital of Athens "G. Gennimatas", Athens, GRC

**Keywords:** paediatric spine surgery, spine deformity surgery, orthopaedics surgery, remote cerebellar hemorrhage, pediatric spine surgery, congenital kyphosis

## Abstract

Congenital deformities of the spine lead to an imbalance in the longitudinal growth of the spine. These growth abnormalities may lead to three main patterns of deformity: scoliosis (the most common), kyphosis or lordosis (the least common). Despite the recent improvements in imaging and the routine use of neuromonitoring in the surgical treatment of congenital kyphosis, this surgery may be associated with a high rate of complications such as neurologic deficit, pulmonary thromboembolic events, infection, deep vein thrombosis, implant failure, and dural injury. In this paper, we report a rare yet devastating complication to raise awareness about patients who have unexpected neurological deterioration after spinal surgery. Early recognition of remote cerebellar haemorrhage (RCH) symptoms is crucial since rapid diagnosis and management lead to a favourable outcome for this potentially life-threatening complication. To our knowledge, this is the first reported case in children.

## Introduction

Congenital deformities of the spine lead to an imbalance in the longitudinal growth of the spine. The condition has been classified into three main types: first, the failure of formation (type I), second, the failure of segmentation (type II), and lastly, a combination of defects of both formation and segmentation (type III) of the vertebral body. There are approximately 10% of patients whose radiological features do not fall into any of the above categories and have been termed type IV unclassified patients [1]. These growth abnormalities may lead to three basic patterns of deformity which are scoliosis, which is the most common, and kyphosis or lordosis, which is the least common [2].

Although much less common than congenital scoliosis, congenital kyphosis poses a significantly greater risk of neurological complications, including spinal cord compression and dural tears [3]. The kyphotic apex can occur at all vertebral levels, but according to McMaster et al., it is most frequently found between T10 and L1 vertebrae [1].

The mainstay of treatment for congenital kyphosis is resection of the hemivertebra and a short spinal fusion. The most common surgical options are the traditional two-stage anterior resection and posterior fixation technique or a single-stage posterior vertebral column resection (VCR) and spinal fusion. In any case, the milestones for successful management are early diagnosis and prompt surgical intervention. Most studies suggest surgical treatment after the patient has turned one year old [4].

The successful outcome of surgical treatment for paediatric kyphosis depends on careful pre-operative evaluation of the patient and the identification of any associated comorbidities that may make the surgical reconstruction more challenging.

Despite the recent improvements in imaging and the broad use of intra-operative neuromonitoring, the surgical treatment of congenital kyphosis may be associated with a high rate of complications such as neurologic deficit, pulmonary thromboembolic events, infection, deep vein thrombosis, implant failure, and dural injury [5]. In this paper, we report a rare complication of remote cerebellar haemorrhage (RCH) after the correction of congenital kyphosis in a child.

## Case presentation

A five-and-a-half-year-old female patient was scheduled in our department for elective surgery for the treatment of congenital thoracolumbar kyphosis with an angle of 70 degrees.

**Figure 1:**
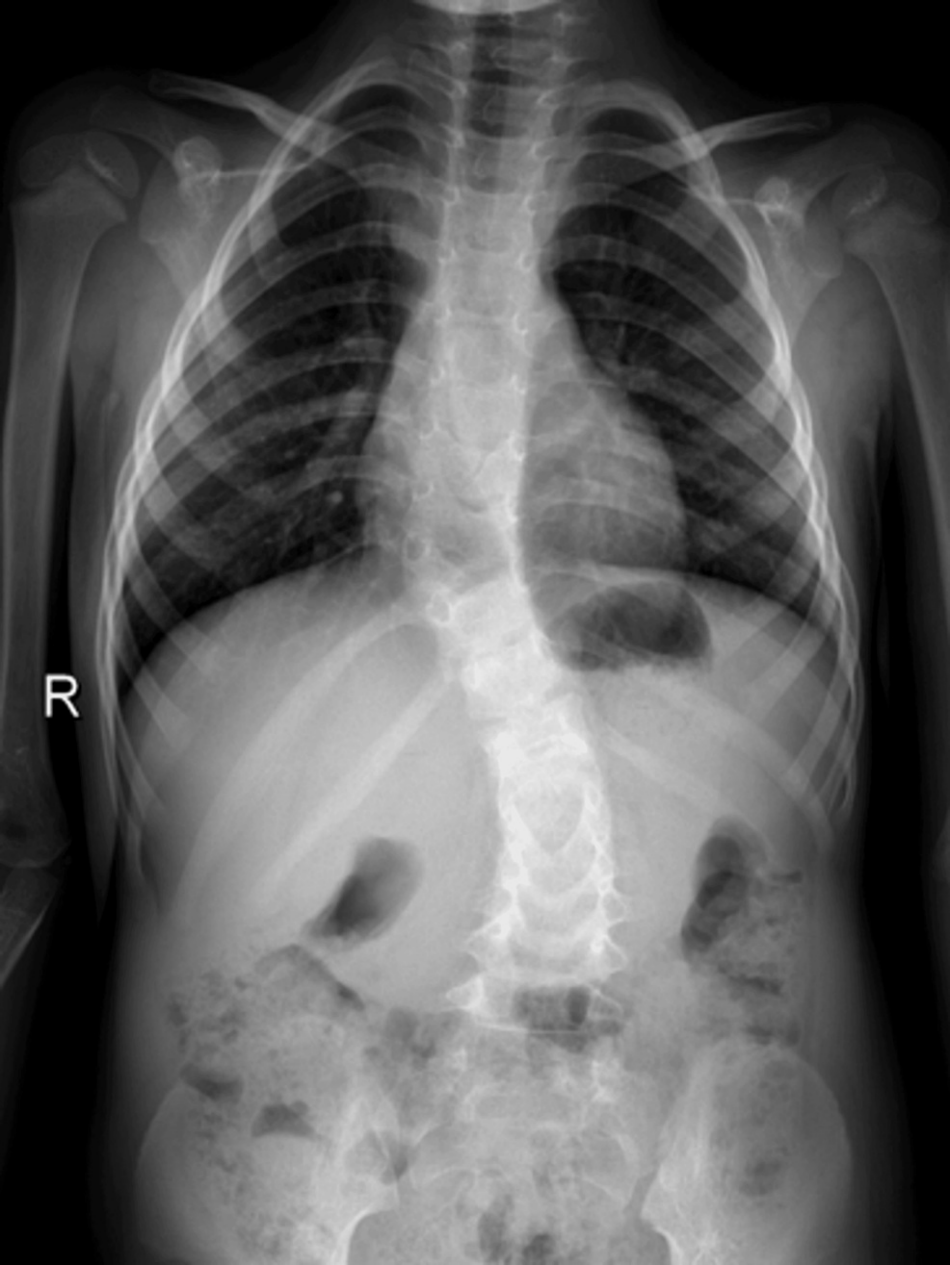
A pre-operative anteroposterior radiograph of the thoracolumbar spine depicts the 70-degree angle of congenital kyphosis and the two hemivertebrae of T10 and T12.

**Figure 2:**
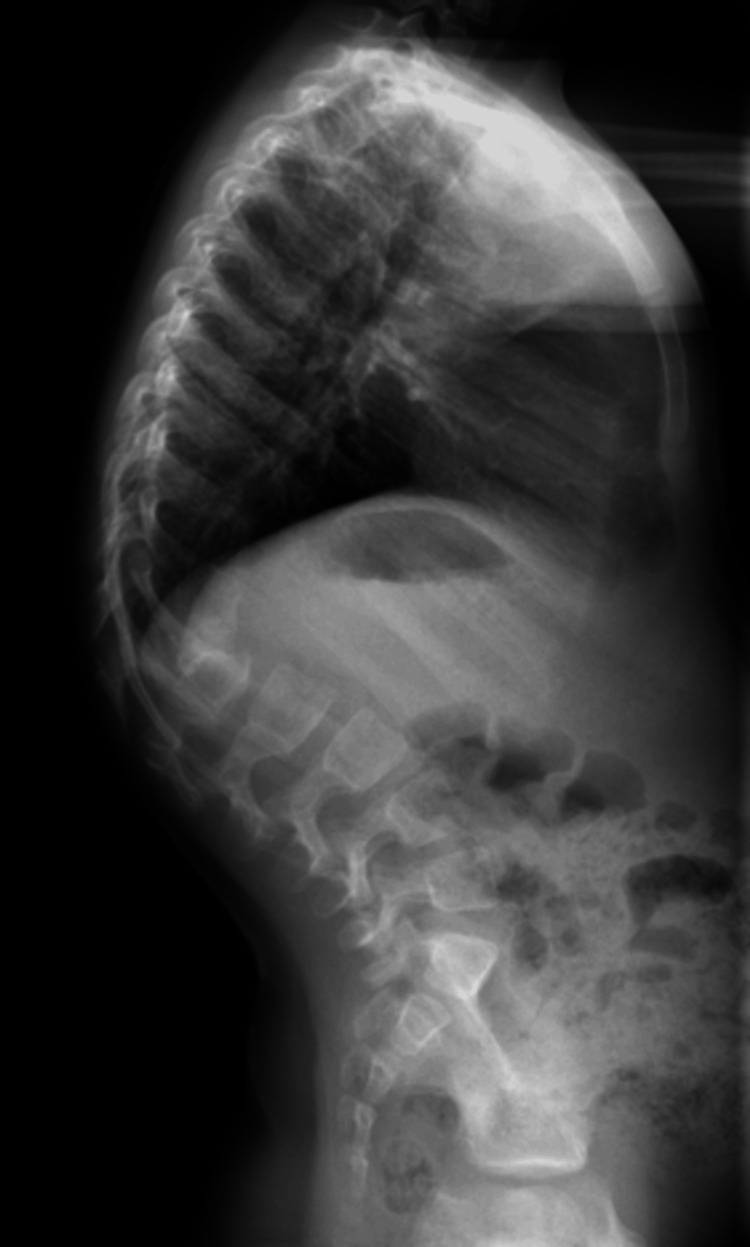
A pre-operative lateral radiograph of the thoracolumbar spine depicts the 70-degree angle of congenital kyphosis and the two hemivertebrae of T10 and T12.

**Figure 3:**
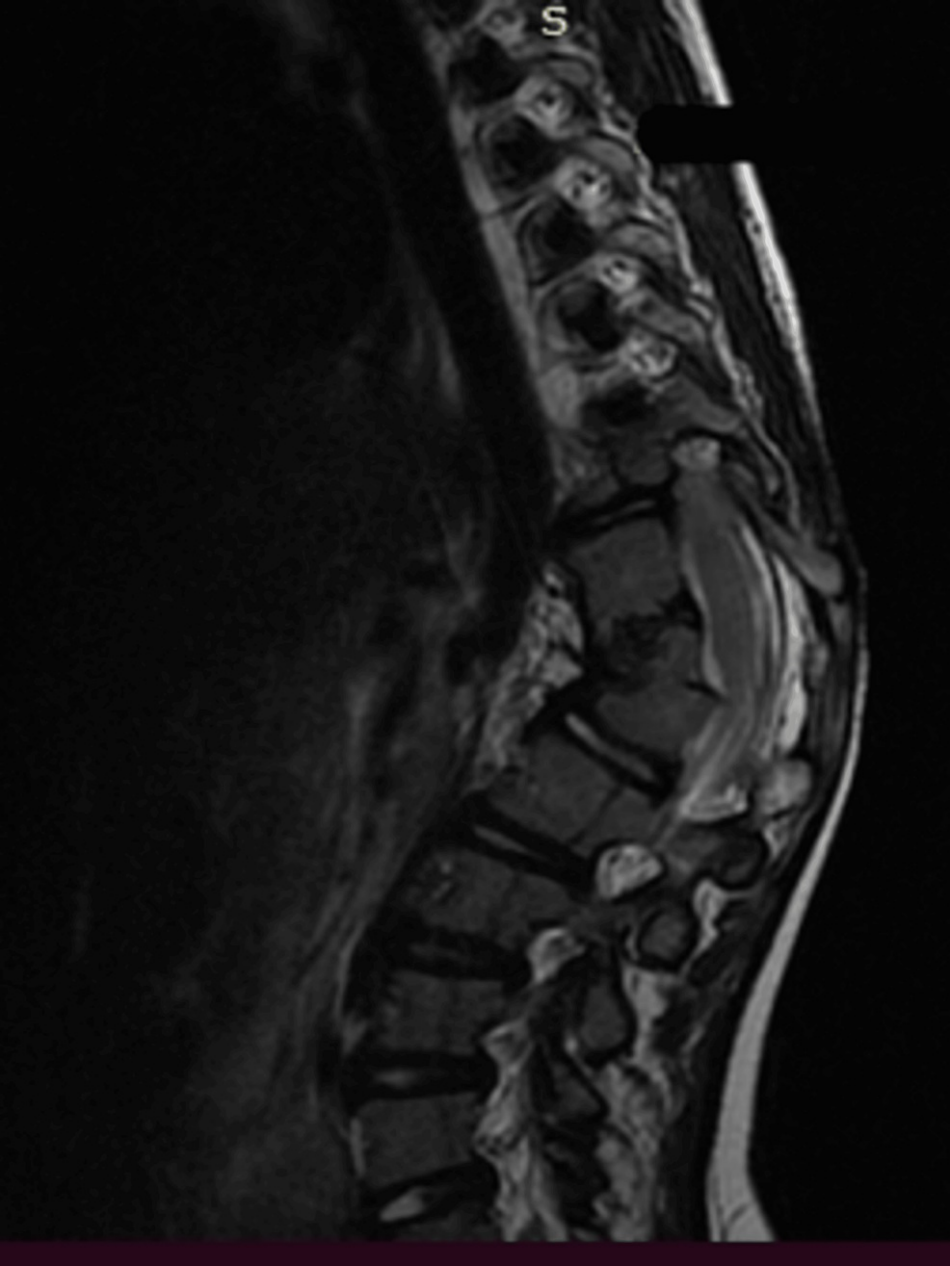
A pre-operative MRI of the thoracolumbar spine depicts the kyphosis level and the hemivertebrae.

**Figure 4:**
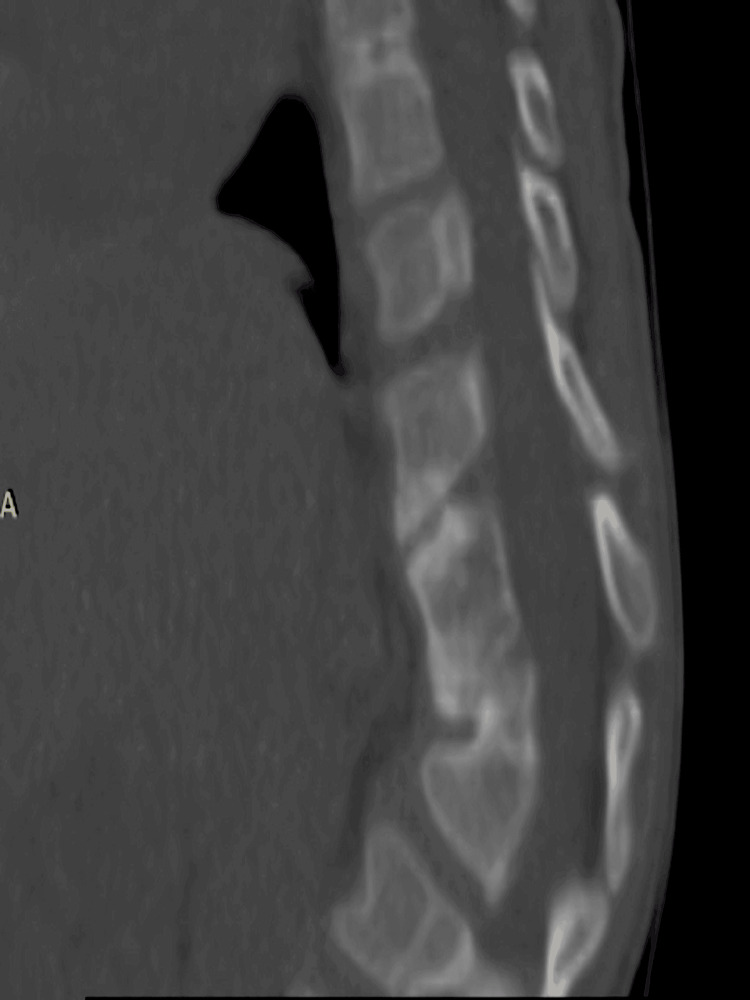
A pre-operative CT of the thoracolumbar spine depicts the kyphosis level and the hemivertebrae.

Two hemivertebrae were present, one on the T10 level, leading to scoliosis, and one on the T12 level, causing kyphosis. (Figures 1-4). No other comorbidities were identified in the specific patient. After meticulous pre-operative evaluation, we decided to address the kyphotic deformity with an all-posterior approach consisting of T12 hemivertebra resection and short spinal fusion between T9 and L2 levels. The procedure took place with the patient in the prone position, under general anaesthesia and continuous electrophysiologic monitoring.

**Figure 5:**
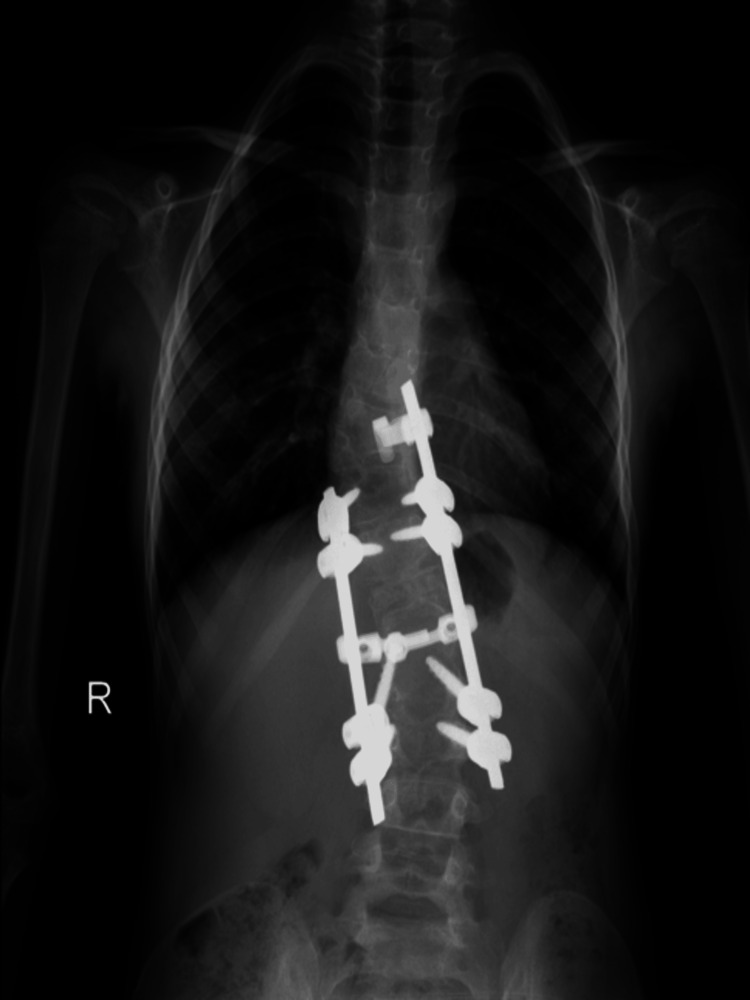
A post-operative anteroposterior radiograph of the thoracolumbar spine depicts the T10-L2 spinal fusion with pedicle screws, the T9 hook, and the resection of T12.

**Figure 6:**
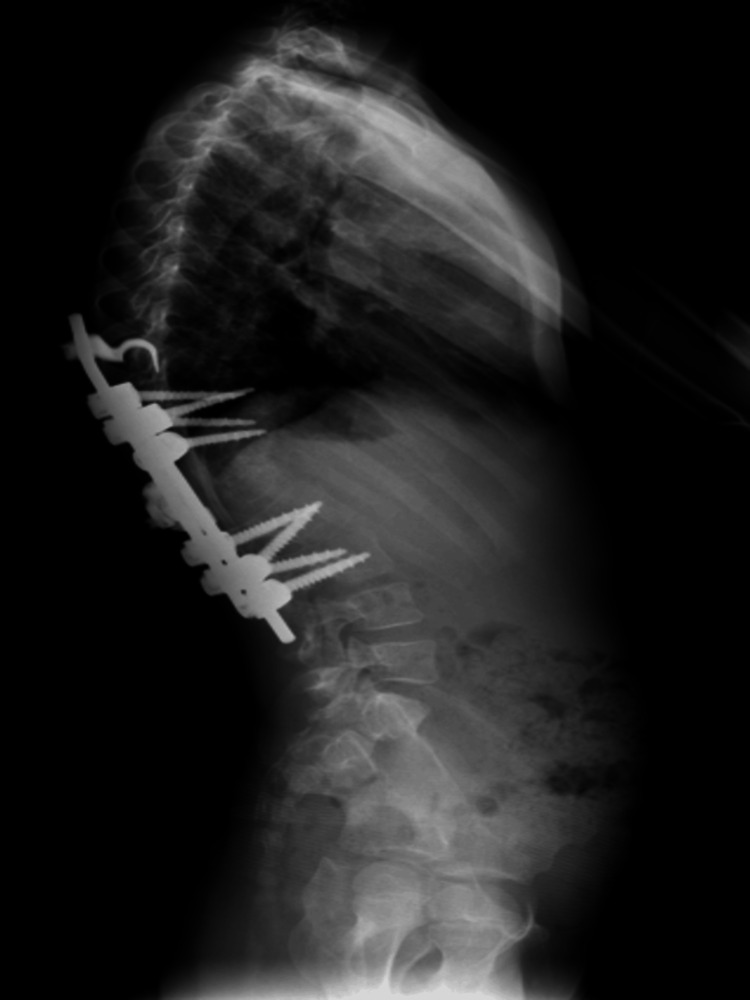
A post-operative lateral radiograph of the thoracolumbar spine depicts the T10-L2 spinal fusion with pedicle screws, the T9 hook, and the resection of T12.

At first, a midline-posterior approach was carried out. All the soft tissues were stripped, and the entry points for the pedicle screws were identified. Pedicle screws were positioned on both sides of the T10, T11, L2, and L3 vertebrae, along with a supralaminar hook on the T9 vertebra. Next, the facets of the hemivertebra and adjacent vertebrae were removed, and a wide laminectomy at the hemivertebra level was performed, exposing the neural structure. The 12th left rib head was removed with cautious surgical preparation and a pleura pushback to access the lateral and anterior portions of the spine. The anterior portion of the hemivertebra was exposed by blunt dissection, and then the vertebral body resection was performed using burrs and curettes of different sizes. Bipolar and electrocauteries were used to maintain hemostasis. Intra-operatively, a dural tear of 2-3 mm took place on the T12 level, which was urgently sutured. Finally, correction of the deformity was performed with two rods via the cantilever method (Figures 5, 6).

**Figure 7:**
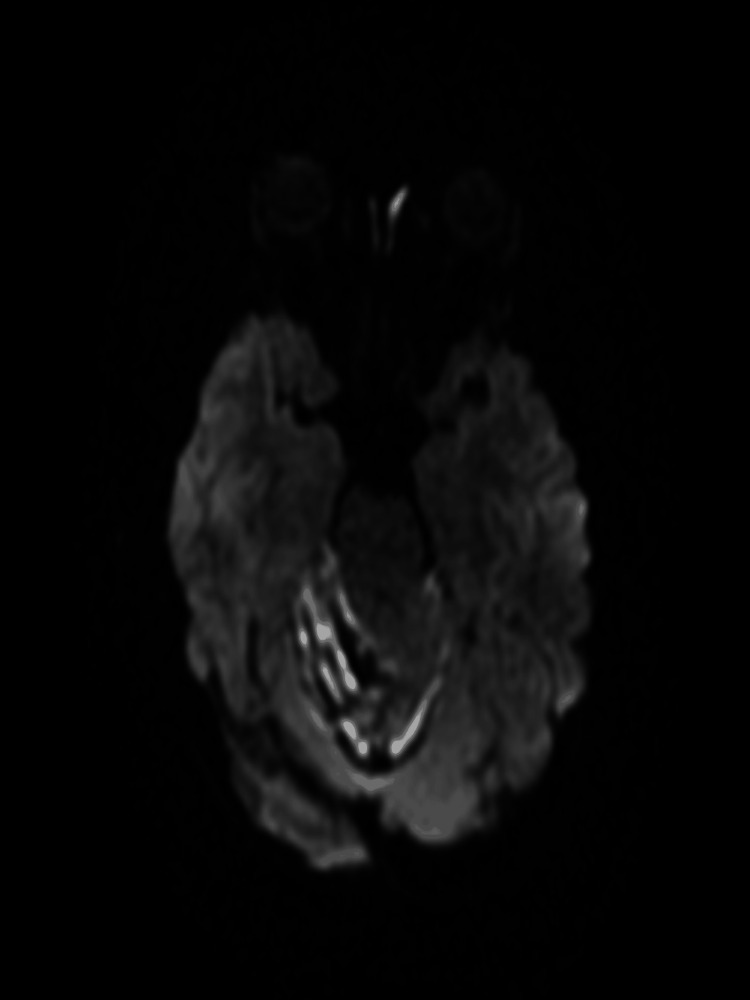
The brain MRA depicts the thrombosis of the cerebellar sigmoid sinus. MRA: magnetic resonance angiogram

**Figure 8:**
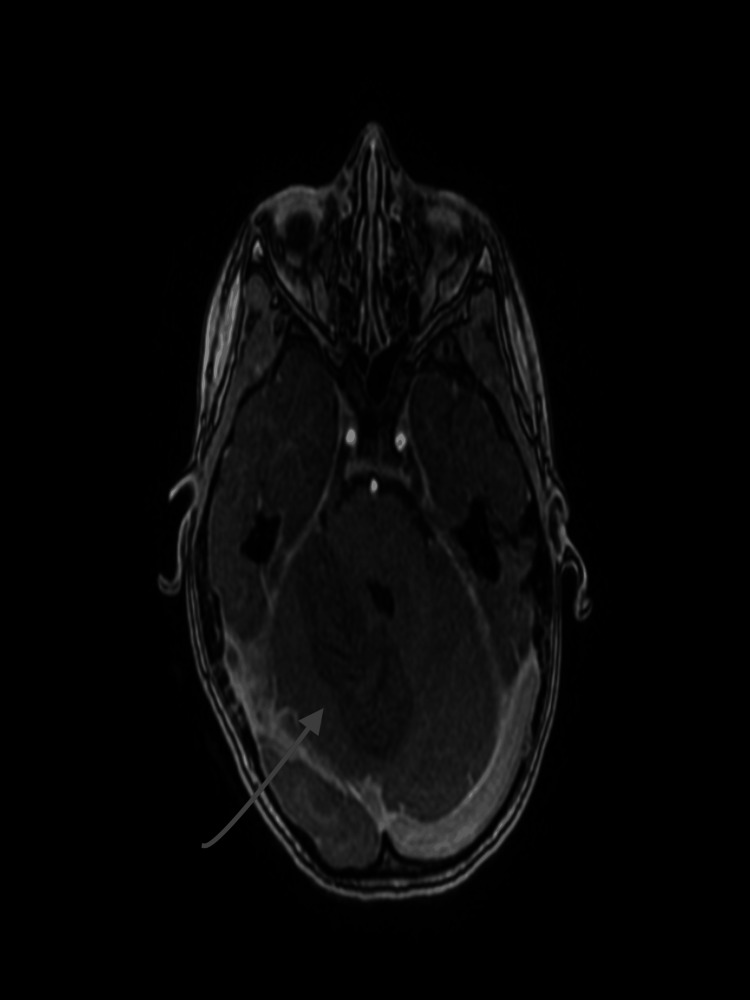
The brain MRA depicts the thrombosis of the cerebellar sigmoid sinus. MRA: magnetic resonance angiogram

At the end of the operation, one surgical drainage was positioned, while a radiographic examination was also completed to confirm proper screw and rod placement. The electrophysiological monitoring was unremarkable by the end of the surgery. The patient was then transferred hemodynamically stable to the ICU for further post-operative care, neurologically intact and with full consciousness. On the second post-operative day, the child developed an episode of loss of consciousness, headache, nausea, and posture imbalance. Urgent brain MRI-magnetic resonance angiogram (MRA) revealed several hemorrhagic infarctions in the cerebellar vermis (Figures 7, 8). 

**Figure 9:**
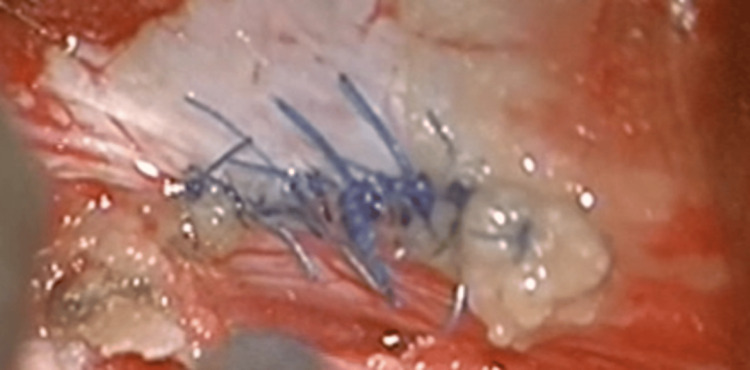
Intra-operative microscope image of the dural tear suturing, also sealed with allograft.

On the third post-operative day, the patient returned to the OR due to increased lucent fluid drainage from surgical trauma. At the re-operation, a dural tear was again revealed on the thoracic level. Suturing was performed using the microscope in collaboration with the neurosurgical team of our hospital, and the dural tear was also sealed with synthetic allograft (Figure 9).

Without any electrophysiologic discrepancies, the patient returned to the department, marking an uneventful post-operative period. The patient was fully mobilised during the post-operative period, together with everyday physiotherapeutic monitoring.

Additionally, the patient received anticoagulant therapy with tinzaparin, which was named according to the antiXa factor levels due to a concomitant thrombosis of the internal jugular vein. The patient had an unremarkable post-operative period with a complete diminishing of the neurologic symptoms of imbalance and headaches and was discharged from the hospital. On the last follow-up, one year post-operatively, she was fully mobilised without any neurological impairment; however, she is still under neurological monitoring on an outpatient basis.

## Discussion

Remote cerebellar haemorrhage is not a common complication of spinal surgical procedures, consisting of approximately 0.08%-0.6% as reported in the current literature, first reported in 1981 [6-8]. Surgical instrumentation of the lumbosacral spine led most frequently to post-operative RCH [9]. However, as reported by Wang et al., even less invasive procedures, such as repeated lumbar punctures, are likely to cause RCH [10]. As highlighted by Sturiale et al., intentional or accidental intra-operative dural tearing was overall reported in about 93% of patients who developed post-operative RCH [9]. The existence of subfascial drainage is described in more than 40% of cases of post-operative RCH, representing a potential risk factor favouring prolonged cerebrospinal fluid (CSF) leakage.

Most of the RCH cases are attributed to venous hemorrhagic infarction. However, the exact pathophysiologic pathway remains unsolved [11]. It is widely proven that RCH likely occurs due to rapid peri-operative excess CSF loss, leading to rapid post-operative hemorrhagic venous infarction. This CSF loss may lead to a so-called “cerebellar sag,” with a cerebellar shift downward; subsequent stretching of the cerebellum may cause venous transient occlusion, leading to both infarction and haemorrhage [12]. The widely called "zebra" sign is the most common bleeding pattern caused by blood spreading in the cerebellar sulci, followed by parenchymal hematoma and mixed haemorrhage, as seen radiographically [13].

Regarding the symptomatology of RCH, headache and impaired consciousness may be present, although nausea, emesis, dysarthria, seizure, diplopia, and even rapid progression towards a comatose state may take place, starting from 10 to 72 hours post-operatively [14, 15].

The mean age of patients at the time of diagnosis of the RCH ranges from 57 to 62 years [9,15]. To our knowledge, this is the first reported case in the existing literature of a child suffering RCH after spinal surgery. Regarding the long-term outcome, it is generally good, with more than 50% of cases having either complete recovery or mild residual neurologic symptoms, despite the mortality risk, which is approximately 10%-15% [13].

## Conclusions

Numerous complications have been described regarding the surgical treatment of congenital spine abnormalities. The rate of neurological complications is high in both adult and paediatric patients. In this paper, we report a rare but devastating complication to raise awareness for patients who experience unexpected neurological deterioration following spinal surgery, particularly paediatric patients, as reported. Early detection of RCH symptoms is critical because prompt diagnosis and treatment lead to a better outcome for this potentially fatal complication.

## References

[1] McMaster MJ, Singh H (1999). Natural history of congenital kyphosis and kyphoscoliosis. A study of one hundred and twelve patients. J Bone Joint Surg Am.

[2] Winter RB, Lonstein JE, Boachie-Adjei O (1996). Congenital spinal deformity. Instr Course Lect.

[3] Winter RB, Moe JH, Wang JF (1973). Congenital kyphosis. Its natural history and treatment as observed in a study of one hundred and thirty patients. J Bone Joint Surg Am.

[4] Klemme WR, Polly DW Jr, Orchowski JR (2001). Hemivertebral excision for congenital scoliosis in very young children. J Pediatr Orthop.

[5] Zhang Z, Wang H, Zheng W (2017). Compressive myelopathy in congenital kyphosis of the upper thoracic spine: a retrospective study of 6 cases. Clin Spine Surg.

[6] Baeesa SS (2012). Remote cerebellar hemorrhage in neurosurgery. Neurosciences (Riyadh).

[7] Brockmann MA, Groden C (2006). Remote cerebellar hemorrhage: a review. Cerebellum.

[8] Chadduck WM (1981). Cerebellar hemorrhage complicating cervical laminectomy. Neurosurgery.

[9] Sturiale CL, Rossetto M, Ermani M (2016). Remote cerebellar hemorrhage after spinal procedures (part 2): a systematic review. Neurosurg Rev.

[10] Wang HY, Hu Z, Han J, Wang D, Wu Q (2023). Remote cerebellar hemorrhage following repeated lumbar punctures. BMC Neurol.

[11] Yasargil MG, Yonekawa Y (1977). Results of microsurgical extra-intracranial arterial bypass in the treatment of cerebral ischemia. Neurosurgery.

[12] Friedman JA, Piepgras DG, Duke DA (2001). Remote cerebellar hemorrhage after supratentorial surgery. Neurosurgery.

[13] Papanastassiou V, Kerr R, Adams C (1996). Contralateral cerebellar hemorrhagic infarction after pterional craniotomy: report of five cases and review of the literature. Neurosurgery.

[14] Koh EJ, Park JS (2017). Fatal remote cerebellar hemorrhage after supratentorial unruptured aneurysm surgery in patient with previous cerebellar infarction: a case report. Medicine (Baltimore).

[15] Numaguchi D, Wada K, Yui M, Tamaki R, Okazaki K (2019). Incidence of remote cerebellar hemorrhage in patients with a dural tear during spinal surgery: a retrospective observational analysis. Spine Surg Relat Res.

